# Betulin exhibits anti-inflammatory activity in LPS-stimulated macrophages and endotoxin-shocked mice through an AMPK/AKT/Nrf2-dependent mechanism

**DOI:** 10.1038/cddis.2017.39

**Published:** 2017-05-18

**Authors:** Xinxin Ci, Junfeng Zhou, Hongming Lv, Qinlei Yu, Liping Peng, Shucheng Hua

**Affiliations:** 1 Institute of Translational Medicine, Department of Respiratory Medicine, The First Hospital, Jilin University, Changchun 130001, China; 2 Department of Dermatology and Venereology, The First Hospital of Jilin University, Changchun, Jilin Province, China

## Abstract

Continued oxidative stress can lead to chronic inflammation, which in turn could mediate most chronic diseases including cancer. Nuclear factor erythroid 2-related factor (Nrf2), a critical transcriptional activator for antioxidative responses, has envolved to be an attractive drug target for the treatment or prevention of human diseases. In the present study, we investigated the effects and mechanisms of betulin on Nrf2 activation and its involvement in the lipopolysaccharide (LPS)-triggered inflammatory system. In macrophages, betulin activated the Nrf2 signaling pathway and increased Nrf2-targeted antioxidant and detoxifying enzymes, including NADPH, quinine oxidoreductase 1 (NQO1), heme oxygenase-1 (HO-1), *γ*-glutamyl cysteine synthetase catalytic subunit (GCLC) and modifier subunit (GCLM) in a dose and time dependent manner. Importantly, we found betulin-induced activation of Nrf2 is AMPK/AKT/GSK3*β* dependent, as pharmacologically inactivating AMPK blocked the activating effect of betulin on AKT, GSK3*β* and Nrf2. Furthermore, betulin attenuated LPS-induced inflammatory mediators (iNOS and COX-2) and MAPK inflammatory signaling pathway. The effect of betulin on HO-1 and NQO1 upregulation, iNOS and COX-2 the downregulation, and survival time extension was largely weakened when Nrf2 was depleted *in vitro* and *in vivo*. Our results demonstrate that the AMPK/AKT/Nrf2 pathways are essential for the anti-inflammatory effects of betulin in LPS-stimulated macrophages and endotoxin-shocked mice.

Oxidative stress is a condition in which generation of reactive oxygen species (ROS) exceeds the capacity of the antioxidant defense system. Extensive research during the last two decades has revealed the mechanism by which continued oxidative stress can lead to inflammation, which in turn could mediate most chronic diseases including cancer.^1^ As a major component of bacterial cell walls, LPS activates the toll-like receptor-4 (TLR4) complex on host cells to dramatically increase the levels of reactive oxygen species (ROS) and initiates the systemic inflammatory response that accompanies sepsis.^2^ Correspondingly, inhibiting the production of intracellular ROS can suppress intracellular proinflammatory signals.^3^ Therefore, modulators of redox balance, considered the key regulators of inflammatory responses, and the antioxidant defense system have become major targets for inflammation research.

Nuclear factor erythroid 2-related factor 2 (Nrf2) is a transcription factor that regulates an adaptive cellular defense response to various stresses, including oxidative, proteotoxic, and metabolic stresses, as well as inflammation.^4^ It plays an imperative role in cellular redox homeostasis and coordinated induction of over 250 genes, including those encoding antioxidant and phase 2 detoxifying enzymes and related proteins, such as NADPH, quinine oxidoreductase 1 (NQO1), heme oxygenase-1 (HO-1), *γ*-glutamyl cysteine synthetase catalytic subunit (GCLC) and modifier subunit (GCLM).^5^ Activating this pathway is one of the main defense mechanisms against oxidative stress.^6^ Nrf2 is held in the cytoplasm as an inactive complex bound to Keap1, a repressor molecule that facilitates Nrf2 ubiquitination.^7^ Although the mechanism by which Nrf2 is liberated from the Keap–Nrf2 complex remains to be established, recent studies have suggested that the phosphorylation of Nrf2 at serine and threonine residues by upstream kinases, such as protein kinase C, phosphatidylinositol-3-kinase/Akt, and mitogen-activated protein kinase, facilitate the release of Nrf2 from Keap1 and its subsequent translocation, followed by the initiation of antioxidative cascades.^8^ AMP-activated protein kinase (AMPK), a heterotrimeric serine/threonine kinase, is an important energy sensor of cellular metabolism in response to metabolic stress, such as oxidative stress, inflammation, and neurodegeneration.^9, 10^ Accordingly, AMPK activation exerts anti-inflammatory effects, regulates catabolism and anabolism and improves redox balance.^11, 12, 13, 14^ Lee and Kim^15^ recently demonstrated that AMPK can stimulate the Nrf2 signaling pathway in Raw264.7 cells, but the underlying mechanisms of the AMPK-mediated antioxidant response remain unclear. Previous studies have suggested that AMPK activated the PI3K/Akt signaling pathway.^16^ Moreover, AMPK also increases inhibitory phosphorylation of glycogen synthase kinase 3 beta (GSK3*β*),^17^ which contributes to mitochondrial protection against iron-induced oxidant stress.^18^ In addition, several lines of evidence have identified GSK3*β* as a novel regulator of Nrf2, which suggests that Nrf2 may cooperate with the AMPK/Akt/GSK3*β* signaling networks.^19, 20^

Betulin (lup-20(29)-ene-3*β*, 28-diol), a triterpene extracted from birch tree bark, has been reported to have diverse pharmacological activities, such as anti-inflammatory, antibacterial and antiviral activities.^21^ Although betulin may exhibit protective effects on cognitive decline in STZ-induced diabetic rats through the HO-1/Nrf-2 pathway and inhibit alcoholic liver injury via activation of AMPK, no information about the potential for betulin involvement in the interactions and temporal relationship between AMPK and Nrf2 pathway in the inflammatory system exists.^22, 23^ In the present study, utilizing LPS-stimulated macrophages and endotoxin-shocked mice, we aimed to investigate the effects of betulin on inflammatory stress and the functional interaction between Nrf2 and AMPK pathways. Our results suggested that the anti-inflammatory role of betulin depends on Nrf2 activation and is primarily involved with the AMPK/AKT/GSK3*β* pathways in LPS-stimulated macrophages and endotoxin-shocked mice.

## Results

### Effects of betulin on Nrf2 translocation, Keap1 and antioxidant enzyme expression

**Figure 1:**
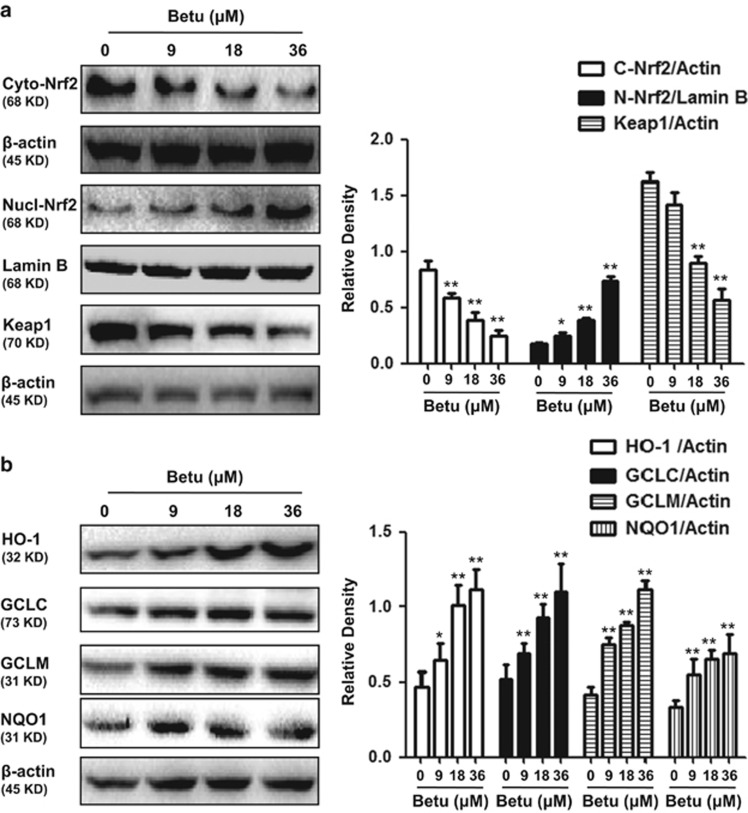
Effects of betulin on Nrf2 translocation, Keap1 and antioxidant enzyme expression. (**a**) RAW 264.7 cells were plated in six-well culture plates and treated with betulin (9, 18 and 36 *μ*M) for 6 h. Nuclear and cytoplasmic extracts from RAW 264.7 cells were prepared for detecting Nrf2 and total protein extracts for detecting Keap1. (**b**) RAW 264.7 cells were treated with betulin (9, 18 and 36 *μ*M) for 18 h and total protein extracts were prepared for detecting antioxidant enzymes. Quantifications were performed by densitometric analysis, and Lamin B and *β*-actin acted as an internal control. A representative western blot is shown in the left panel. Shown in the right panel are means±S.E.M. of three independent experiments. *P≤0.05, **P≤0.01 *versus* the control group

The Keap1-Nrf2 signaling axis is a master regulator that regulates the ARE-driven expression of phase II detoxifying and antioxidant enzymes, such as HO-1, NQO1 and *γ*-glutamate cysteine ligase subunit (GCLS). As shown in Figures 1a and b, treatment with betulin increased Nrf2 translocation from cytoplasm to nucleus and downregulated the expression of the Keap1 protein in a dose-dependent manner. Moreover, we further assessed the expression of antioxidant enzymes and demonstrated that HO-1, NQO1,GCLC and GCLM were upregulated by betulin in a dose-dependent manner (Figures 1c and d).

### Effects of betulin on the AMPK/AKT/GSK3*β* and MAPK pathway in RAW 264.7 cells

**Figure 2:**
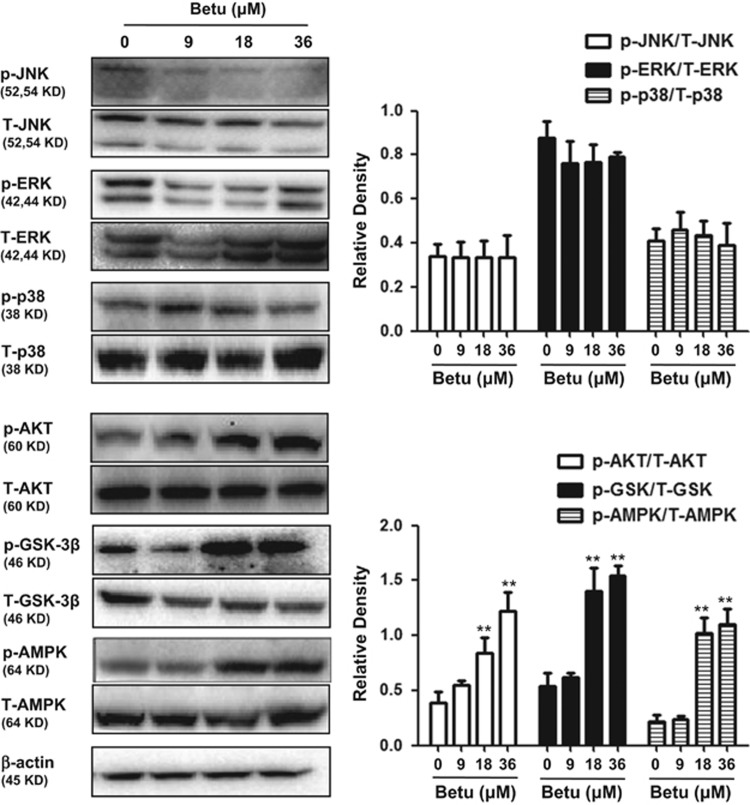
Effects of betulin on AMPK/AKT/GSK3*β* and MAPK pathways in RAW 264.7 cells. RAW 264.7 cells were treated with betulin (9, 18 and 36 *μ*M) for 6 h and then immunoblotted with specific antibodies. Quantifications were performed by densitometric analysis, and *β*-actin acted as an internal control. A representative western blot is shown in the left panel. Shown in the right panel are means±S.E.M. of three independent experiments. **P≤0.01 *versus* the control group

Recent reports have suggested that phosphatidylinositol 3-kinase (PI3K), c-Jun N-terminal kinase (JNK) and extracellular signal-regulated protein kinase (ERK) are assumed to facilitate the release of Nrf2 from Keap1 and its subsequent translocation. We examined the activation of betulin on the AKT and MAPK pathway in RAW264.7 cells. The results indicated that betulin slightly decreased JNK and ERK, slightly increased p38, but significantly increased AKT phosphorylation in a dose-dependent manner (Figure 2). Moreover, previous studies suggested that AMPK activated the PI3K/Akt signaling pathway and increased inhibitory phosphorylation of glycogen synthase kinase 3 beta (GSK3*β*). We examined the effect of betulin on AMPK and GSK3*β* phosphorylation and demonstrated that betulin activated AMPK and GSK3*β* phosphorylation in a dose-dependent manner.

### Involvement of the AMPK/AKT/GSK3*β* pathway in Nrf2 nuclear translocation by Betulin

**Figure 3:**
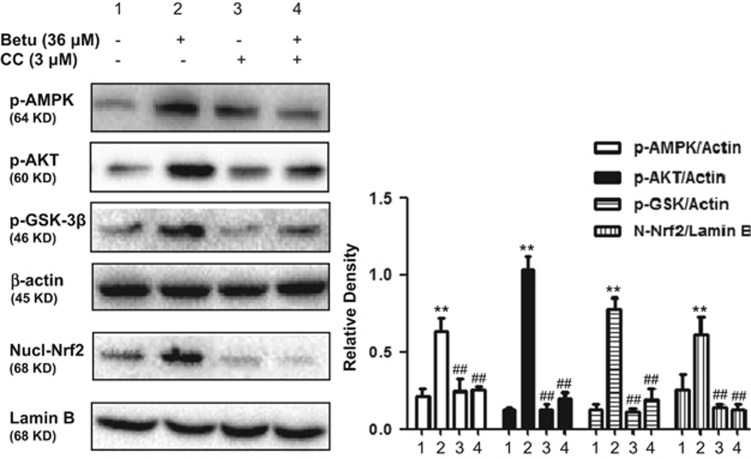
Involvement of the AMPK/AKT/GSK3*β* pathway in Nrf2 nuclear translocation by Betulin. Cells were treated with 3 *μ*M compound C (Comp.C) for 18 h before treatment with betulin (36 *μ*M) for 6 h. Cell lysates were immunoblotted for the phosphorylation of AMPK, Akt, GSK3*β* and Nrf2. A representative western blot is shown in the left panel. Shown in the right panel are means±S.E.M. of three independent experiments. **P≤0.01 *versus* the control group; ^##^*P*≤0.01 *versus* the betulin group

To further determine the upstream signaling pathway involved in betulin-mediated Nrf2 activation, we investigated the effects of compound C, a specific inhibitor of AMPK, on Nrf2 nuclear translocation. As shown in Figure 3, compound C dramatically inhibited AMPK, AKT and GSK3*β* phosphorylation and Nrf2 nuclear translocation, which suggested that betulin modulated *Nrf2* nuclear translocation via the activation of the AMPK/AKT/GSK3*β* signaling pathway in RAW264.7 cells.

### Betulin activated anti-inflammatory and antioxidant reactions in LPS-stimulated macrophages

**Figure 4:**
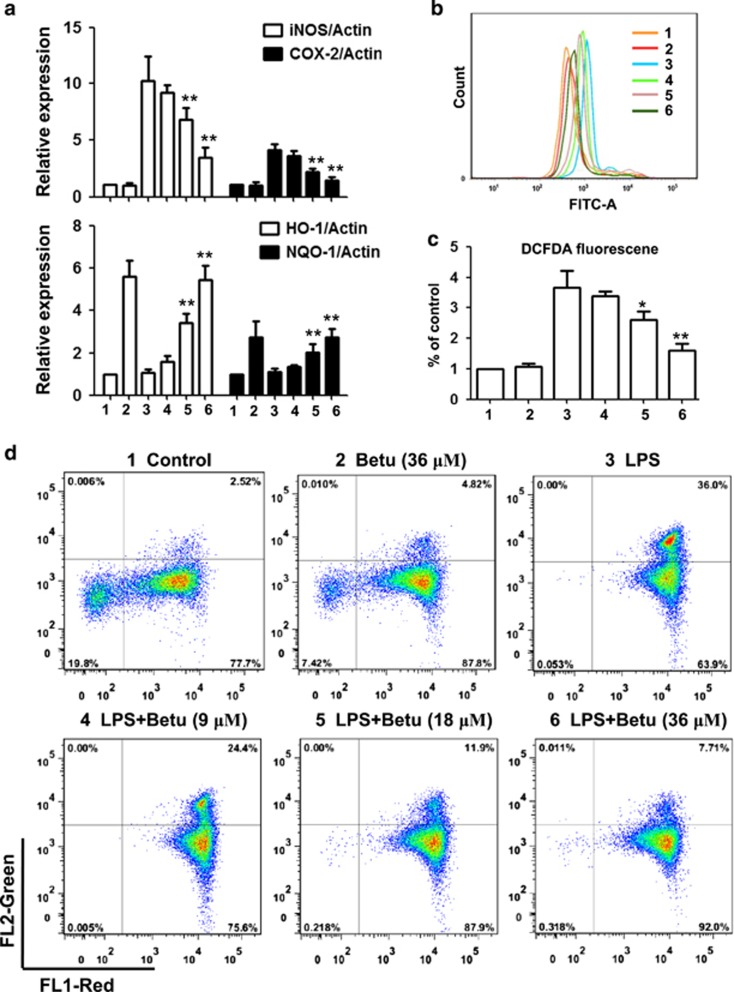
Effects of betulin on anti-inflammatory and antioxidant reactions in LPS-stimulated macrophages. RAW264.7 cells were plated in 12-well plates, preincubated with betulin (9, 18 and 36 *μ*M) for 1 h, and then challenged with LPS (500 ng/ml) for 18 h. (**a**) The expression of proinflammatory genes (iNOS and COX-2) and antioxidant enzymes (HO-1 and NQO1) were detected by quantitative real-time PCR. (**b** and **c**) The cells were incubated with 50 *μ*M of DCFH-DA for 40 min, and DCF fluorescence intensities were detected by flow cytometry. (**d**) The cells were washed with PBS and incubated with JC-1 (10 *μ*g/ml) at 37 °C in the dark for 30 min. JC-1 fluorescence was quantified through flow cytometry. *P⩽0.05, **P⩽0.01 versus the LPS group

Elevation of inflammatory gene expression is a well-known response of macrophages to LPS stimulation; therefore, we assessed the suppressive effect of betulin on the representative inflammatory genes. As shown in Figure 4a, betulin significantly suppressed LPS-induced expression of iNOS and COX-2. Furthermore, Nrf2-regulated antioxidant enzymes play a vital role in the anti-inflammatory response. As shown in Figure 4a, LPS had almost no effect on the expression of HO-1 and NQO1, but betulin significantly upregulated expression of these genes. Furthermore, a burst of production of free radicals is known to accompany the inflammatory response; therefore we detected the effect of betulin on ROS production. As shown in Figures 4b and c, betulin alone did not affect ROS production, but significantly inhibited LPS-induced ROS production. Moreover, protecting mitochondria from free radicals is an important aspect of the antioxidative and anti-inflammatory responses. To address the effect of betulin on mitochondrial protection, we first assayed mitochondrial membrane potential using a fluorescent mitochondrial dye, JC-1. As shown in Figure 4d, MMP was greatly degraded by LPS, showing obvious green fluorescence, while betulin effectively restored the MMP.

### Knockout of Nrf2 abolished the protective effects of betulin on inflammatory and oxidant reactions in LPS-stimulated macrophages

**Figure 5:**
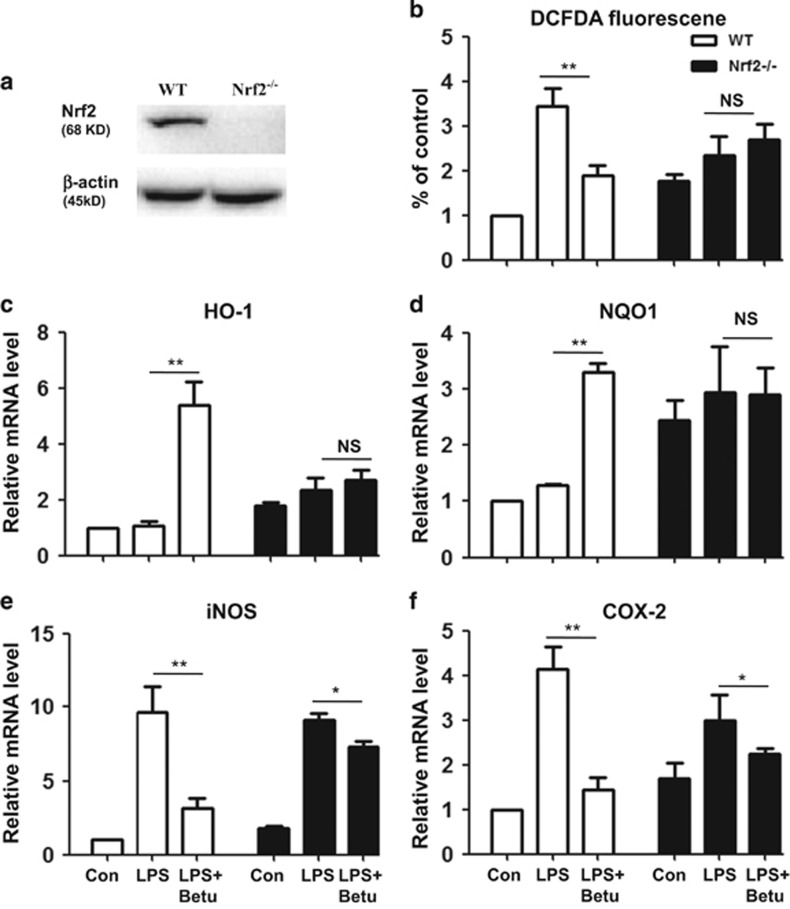
Knockout of Nrf2 abolished the protective effects of betulin on inflammatory and oxidant reactions in LPS-stimulated macrophages. (**a**) Normal (WT) and Nrf2 knockout (Nrf2^−/−^) RAW264.7 cells were cultured with betulin (36 *μ*M) for 6 h to detect Nrf2 expression. (**b**) WT and Nrf2^−/−^ cells were cultured with or without betulin (36 *μ*M) for 1 h and then challenged with LPS (500 ng/ml) for 18 h; then, the cells were incubated with 50 *μ*M of DCFH-DA for 40 min, and DCF fluorescence intensities were detected by flow cytometry. (**c**–**f**), WT cells and Nrf2^−/−^ cells were cultured with or without betulin (36 *μ*M) for 1 h and then challenged with LPS (500 ng/ml) for 18 h; then, the expression of proinflammatory genes (iNOS and COX-2) and antioxidant enzymes (HO-1 and NQO1) were detected by quantitative real-time PCR. All of the data shown represent the average of three independent experiments. *P≤0.05, **P≤0.01 *versus* the LPS group; NS, no significant difference

Antioxidation is important for the anti-inflammatory response, with Nrf2-regulated antioxidant enzymes being vital to this antioxidative response. Therefore, we used RAW264.7 Nrf2^−/−^ cells to investigate whether the anti-inflammatory and antioxidant effects of betulin were mediated by Nrf2. Compared with control cells, Nrf2^−/−^ cells markedly suppressed the Nrf2, HO-1 and NQO1 protein expression induced by betulin (Figures 5a, c and d). Furthermore, our results showed that the protective effects of betulin on iNOS, COX-2 and ROS production were attenuated in Nrf2^−/−^ cells (Figures 5b, e and f).

### Betulin regulated anti-inflammatory and antioxidant signaling pathways in LPS-stimulated macrophages

**Figure 6:**
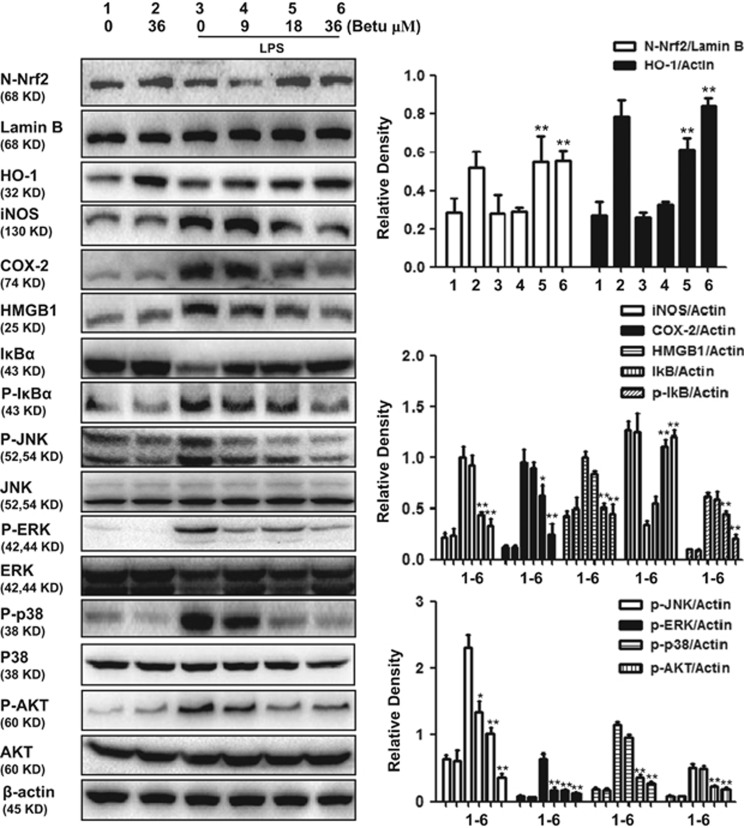
Effect of betulin on anti-inflammatory and antioxidant signaling pathways in LPS-stimulated macrophages. RAW264.7 cells were plated in 6-well plates, preincubated with betulin (9, 18 and 36 *μ*M) for 1 h, and then challenged with LPS (500 ng/ml) for 18 h. Cell lysates were immunoblotted with different antibodies. A representative Western blot is shown in the left panel. Shown in the right panel are means±S.E.M. of three independent experiments. *P≤0.05, **P≤0.01 *versus* the LPS group

Nrf2 translocation from the cytoplasm to the nucleus is a dispensable step for Nrf2 activation. The nuclear distribution of Nrf2 protein was determined by western-blot assay, with the results showing that LPS has no effect on Nrf2 protein in the nuclear fraction, but that betulin alone or together with LPS increased nuclear Nrf2 protein expression (Figure 6). HO-1, an antioxidant enzyme regulated by Nrf2, was shown to be upregulated by betulin alone or together with LPS. Some typical signaling pathways, such as MAPK, NF-*κ*B and AKT, are involved in the inflammatory response; therefore, we analyzed the effects of betulin on LPS-stimulated inflammatory protein expression. Our results indicated that the protein expression of iNOS, COX-2 and HMGB1 and the levels of JNK, ERK, p38 and AKT phosphorylation dramatically increased in RAW264.7 cells exposed to LPS, whereas the expression of all of these proteins decreased following betulin pretreatment.

### Suppressive effects of betulin on LPS-induced endotoxin shock is Nrf2 dependent in mice

**Figure 7:**
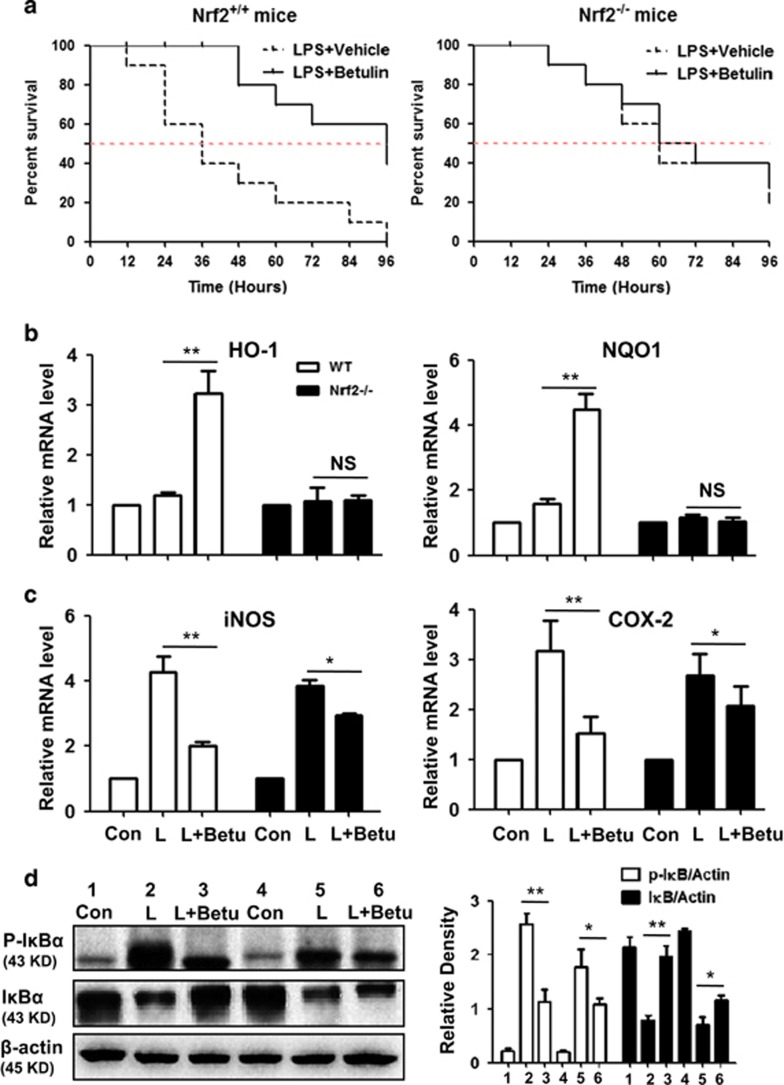
Suppressive effects of betulin on LPS-induced endotoxin shock are Nrf2 dependent in mice. (**a**) Effect of betulin on the survival rate of wt or Nrf2^−/−^mice. Mice were treated with betulin (12 mg/kg) and LPS (40 mg/kg) as described in the Material and Methods (*n*=10); the survival rates are presented as survival curves. (**b**–**d**), Effect of betulin on the genes and protein expression in the lung tissue of mice. P*≤0.05, P^**^≤0.01 *versus* the LPS group; NS, no significant difference

The dependency of the anti-inflammatory role of betulin on Nrf2 was further assessed in endotoxin-shock mice. First, as shown in Figure 7a, for Nrf2^+/+^ mice (WT), the median survival times in the control group and in the betulin-treated group were 36 and 96 h, respectively, suggesting that betulin has significant protective effects. However, for Nrf2^−/−^ mice, the median survival times both in the control group and in the betulin-treated group were 60 h. Second, for WT mice, the final survival rate was 0% (control group) *versus* 40% (betulin-treated group). Meanwhile, for Nrf2^−/−^ mice, the final survival rate was 20% (control group) *versus* 30% (betulin-treated group). Since lung injury is remarkable in LPS-shocked mice, we isolated RNA from lung tissue. As shown in Figures 7b–d, betulin significantly increased mRNA expression of antioxidant genes (HO-1 and NQO1), decreased the mRNA expression of anti-inflammatory genes (iNOS and COX-2) and decreased I*κ*B*α* phospholation. However, both the antioxidant and anti-inflammatory effects of betulin were abrogated or attenuated in Nrf2^−/−^ mice.

## Discussion

Extensive research during the last two decades has revealed that oxidative stress can activate inflammatory signaling pathways such as NF-*κ*B and MAPK, which regulate cytokines, chemokines, cyclooxygenase-2 (COX-2), inducible nitric oxide synthase (iNOS), and anti-inflammatory molecules.^24^ Continued oxidative stress can lead to inflammation, which can further mediate most chronic diseases including cancer, diabetes, cardiovascular and pulmonary diseases.^25^ The nuclear factor erythroid 2-related factor 2 (Nrf2) pathway plays an imperative role in cellular redox homeostasis, and the activation of this pathway is one of the main defense mechanisms against oxidative stress. In the present study, we found that betulin has anti-inflammatory potential via its activation of the Nrf2 pathway, and this activation of Nrf2 is AMPK-dependent in a LPS-challenged inflammatory response.

Nrf2 can prevent cellular damage associated with various types of injury in many different cell types. Nrf2-mediated protection depends on the expression of detoxification and antioxidant genes such as HO-1, NQO1, GCLC and GCLM.^5^ Previous studies also link the activation of PI3K, PKC and MAPK signaling pathways to the regulation of Nrf2-related antioxidant gene expression.^26^ In the present study, we demonstrated that betulin regulation of Nrf2 and antioxidant gene expression is mediated through degradation of Keap1 and activation of Akt and AMPK; pretreatment with AMPK spefific inhibitors (Compound C) attenuated the activation of AKT, Akt-mediated inhibitory phosphorylation of GSK3*β* and consequently Nrf2 which induced by betulin. Taken together, these results suggested that betulin has antioxidant potential via its activation of the AMPK/AKT/GSK3*β*/Nrf2 and Nrf2/Keap1 pathways in RAW264.7 cells.

Many studies have reported on the therapeutic benefits of betulin in experimental animal models. The most widely studied effect of betulin is its anti-inflammatory activity in various inflammatory model including COPD, alcoholic liver injury, mammary gland inflammatory injury and chronic dermal inflammation.^23, 27, 28^ It has been reported that betulin decreased proinflammatory cytokines via inhibiting NF-*κ*B and MAPK signaling pathways.^29^ Given this situation and the promising importance of the relationship between oxidative stress and inflammation, we posed two specific questions: is there a functional link between the anti-inflammatory and antioxidant effects of betulin? If there is, how do they work together to inhibit inflammation? On the basis of these questions, we assessed the effect of betulin on representative inflammatory genes (iNOS and COX-2) and antioxidant enzymes (HO-1 and NQO1) in LPS-stimulated RAW264.7 cells. Our results demonstrated that betulin significantly suppressed the LPS-induced expression of iNOS and COX-2 but upregulated HO-1 and NQO1 gene expression. Furthermore, LPS-induced ROS production and MMP degradation were significantly inhibited by betulin. These investigations indicated that betulin possessed anti-inflammatory and antioxidant effects, whereas these effects were attenuated or abolished in RAW264.7 Nrf2^−/−^ cells, suggesting that the compensatory upregulation of Nrf2-mediated expression of antioxidant genes is induced by betulin to protect against LPS-induced inflammation.

On the basis of above outcome, we further explored the mechanisms involved in the anti-inflammatory effects of betulin. Several reports have shown that the LPS signaling cascade leading to iNOS and COX2 expression in macrophages is dependent on the phospholation of the members of the MAPKs family: p38, ERK1/2 and NF-*κ*B p65 translocation from the cytoplasm to the nucleus.^30, 31^ Under resting conditions, NF-*κ*B is held inactive by I*κ*B. The phosphorylation of I*κ*B results in I*κ*B degradation and further NF-*κ*B dissociation, which leads to the induction of pro-inflammatory cytokines and mediators.^32^ Protein expression was accordingly measured in western blot assays. Our results indicate that LPS markedly induced the inflammatory protein expression but did not affect Nrf2 and HO-1 expression, which was regulated by betulin pretreatment. We further investigated the Nrf2 pathway involved in the anti-inflammatory effect of betulin against LPS *in vivo*. For Nrf2^+/+^ endotoxin-shocked mice, betulin prevented the mortality associated with LPS challenge, but the anti-inflammatory role of betulin was clearly attenuated in Nrf2-deficient mice. Surprisingly, Nrf2^−/−^ mice were less susceptible to LPS and developed less severe endotoxin-shocked phenotypes than Nrf2^+/+^ mice did, as determined by a slight decrease in inflammation and an increased survival rate. Moreover, consistent with *in vitro* results, betulin abolished the upregulation of HO-1 and NQO1 but only attenuated the downregulation of iNOS and COX-2 expression in the lung tissue of Nrf2^−/−^ mice. In addition, the inhibitory effect of betulin on NF-*κ*B, as a regulator of iNOS and COX-2 expression, has been attenuated in the lung tissue of Nrf2^−/−^ mice. Taken together, our results support that the inhibitory effect of betulin in inflammation is, at least partially, based on a mechanism underlying the suppression of proinflammatory cascades on one hand, and on the activation of Nrf2-mediated antioxidative cascades on the other hand.

In conclusion, our present study for the first time demonstrates that betulin exhibits anti-inflammatory activity in LPS-stimulated macrophages and endotoxin-shocked mice through activation of Nrf2 and that AMPK/AKT/GSK3*β* works upstream of Nrf2. This finding provides an innovative platform to explore the mechanism underlying anti-inflammatory and antioxidant effects and further contributes to the development of new therapeutic approaches for inflammatory diseases.

## Materials and methods

### Reagents

Betulin (analytical grade, purity≥98%) was obtained from the National Institute for the Control of Pharmaceutical and Biological Products (Beijing, China). LPS and LY294002 were purchased from the Sigma Chemical Co. (St. Louis, MO, USA). Compound C (AMPK inhibitor) was obtained from MedChem Express (Monmouth, NJ, USA). Antibodies against Nrf2, HO-1, NQO1, GCLC, GCLM, iNOS, COX-2, HMGB1, KEAP1, AKT, p-AKT, GSK3*β*, p-GSK3*β* Ser,^9^ ERK, pERK, JNK, p-JNK, p38, p-p38, AMPK, p-AMPK*α* (Thr172), I*κ*B*α*, P-I*κ*B*α*, Lamin B, as well as *β*-actin were purchased from Cell Signaling (Boston, MA, USA) or Abcam (Cambridge, MA, USA). The horseradish peroxidase-conjugated anti-rabbit and anti-mouse IgG were purchased from proteintech (Boston, MA, USA). Prime-Script RT-PCR kit and Faststart Universal SYBR Green Master were offered by Takara (Dalian, China) and Roche (Basel, Switzerland), respectively. 3′-tetraethyl-benzimidazolyl-carbocyanine iodide (JC-1) were offered from Beyotime Institute of Biotechnology (Jiangsu, China).

### Cells

The RAW264.7 murine macrophage cell line was obtained from the China Cell Line Bank (Beijing, China). Cells were cultured in Dulbecco’s modified Eagle medium supplemented with 3 mM glutamine, antibiotics (100 U/ml penicillin and 100 U/ml streptomycin), and 10% heat-inactivated fetal bovine serum. The cells were maintained at 37 °C in a humidified incubator containing 5% CO_2_. In all experiments, cells were allowed to acclimate for 24 h before any treatments.

### Animals

Normal B6 mice (Nrf2^+/+^) and Nrf2 deficient mice(Nrf2^−/−^) with B6 background were provided by Beijing weitonglihua and Jackson lab. All animals were bred under specific pathogen-free conditions. All experiments were conducted according to the experimental practices and standards approved by the Animal Welfare and Research Ethics Committee at Jilin University.

### CRISPR/Cas9 knockout

RAW264.7 cells were grown in 24-well plates for 16 h, and then transfected with one plasmid expressing Cas9 with Nrf2 sgRNA and one plasmid carrying a puromycin resistance gene using Viafect transfection reagent (Promega, Madison, USA). After 36 h, 2 mg/ml puromycin was added to selected cells. Two days later, live cells were seeded in 96-well plates at a density of one cell per well. The level of gene editing efficiency after the clonal expansion was determined by immunoblotting. To verify the edited genes, DNA sequencing was employed.

### Quantitative real-time polymerase chain reaction analysis

Cells were treated with or without betulin (9, 18 and 36 *μ*M) for 1 h, then treated with LPS (500 ng/ml) for 18 h. Total RNA from cells was isolated by using Trizol reagent, and the manufacturer’s instructions. After the concentration of RNA was determined by spectrophotometer, 1 *μ*g of RNA was converted to cDNA by Prime-Script RT-PCR kit Real-time PCR analysis was performed using the Applied Biosystems 7300 real-time PCR system and software (Applied Biosystems, Carlsbad, CA, USA). Real-time PCR was conducted in 0.2 ml PCR tubes with forward and reverse primers and the SYBR green working solution, using a custom PCR master mix with the following conditions: 95 °C for 10 min, followed by 40 cycles of 95 °C for 10 s, 60 °C for 30 s. The relative gene expression were analyzed by normalizing with GAPDH mRNA expression.

### Western blot analysis

Cells were treated with or without betulin (9, 18 and 36 *μ*M) for 1 h, then treated with LPS (500 ng/ml) for 18 h. Cell homogenates were centrifuged at 3000 × *g* for 5 min. Nuclear and cytoplasmic fractions of cell were prepared, as previously described.^33^ Whole cell lysates were lysed in 1% nondiet-P40 lysis buffer (1% NP-40, 150 mM Nacl, 50 mM Tris, pH 7.4) with freshly added protease and phosphatase inhibitors. After the lysates were incubated on ice for 30 min, they were centrifuged (12 000 × *g* at 4  °C) for 5 min to obtain the cytosolic fraction. Protein concentrations were determined using the Bradford assay before storage at −80 °C. Cell lysates were subjected to immunoblotting analysis using the following antibodies: anti-pAKT, anti-pGSK3*β*, anti-pAMPK, anti-pJNK, anti-pERK, anti-pp38, anti-AKT, anti-GSK3*β*, anti-AMPK, anti-JNK, anti-ERK, anti-p38, anti-Nrf-2, anti-HO-1, anti-COX-2, anti-iNOS, anti-HMGB1, anti-I*κ*B*α*, anti-pI*κ*B*α*, and anti-*β*-actin. The membranes were further probed with horseradish peroxidase-conjugated secondary antibodies, and detected by ECL western blot substrate. Band intensities were quantified using Image J gel analysis software. The fold increase in the level of protein expression was calculated by comparing it with that of normal controls. The experiments were repeated three times for each experimental condition.

### Intracellular ROS measurement

To detect intracellular ROS production, RAW 264.7 cells were seeded into 12-well plates (4 × 10^5^ cells/well) and treated with or without betulin (9, 18 and 36 *μ*M) for 1 h, then the cells were treated with LPS (500 ng/ml) for 18 h. Next, the cells were incubated with 50 *μ*M of DCFH-DA for 40 min, and DCF fluorescence intensities were detected by flow cytometry.

### JC-1 assay for mitochondrial membrane potential

Mitochondrial membrane potential (MMP) was measured using a mitochondrial-specific dual-fluorescence probe, JC-1 (Beyotime). Briefly, RAW 264.7 cells were seeded into 12-well plates (4 × 10^5^ cells/well), treated with or without betulin (9, 18 and 36 *μ*M) for 1 h, and then treated with LPS (500 ng/ml) for 18 h. Next, the cells were washed with PBS and incubated with JC-1 (10 *μ*g/ml) at 37 °C in the dark for 30 min. JC-1 fluorescence was quantified through flow cytometry, in which red JC-1 aggregates were gated in the FL2 channel and green JC-1 monomers in the FL1 channel.

### Animal experiments

Female mice, were randomly grouped (three groups containing 10 mice) and challenged with LPS (40 mg/kg) by intraperitoneal (i.p.) injection or normal saline as a control, with two i.p. injections of Betulin (12 mg/kg), 1 h before and 12 h after the injection of LPS. Survival rates of animals were monitored every 12 h for 4 days.

### Statistical analysis

Two-tailed unpaired Student’s *t* tests were performed to determine *P*-values. All the graphs represent the mean±S.E.M. of three independent experiments, and the asterisks represent *P*-values: *P<0.05, **P<0.01.
